# Tunable microwave absorption of switchable complexes operating near room temperature†

**DOI:** 10.1039/d0ra02236e

**Published:** 2020-06-05

**Authors:** Olesia I. Kucheriv, Viktor V. Oliynyk, Volodymyr V. Zagorodnii, Vilen L. Launets, Olena V. Penkivska, Igor O. Fritsky, Il'ya A. Gural'skiy

**Affiliations:** Department of Chemistry, Taras Shevchenko National University of Kyiv 64 Volodymyrska St. Kyiv 01601 Ukraine illia.guralskyi@univ.kiev.ua; Department of Radio Physics, Electronics and Computer Systems, Taras Shevchenko National University of Kyiv 64 Volodymyrska St. Kyiv 01601 Ukraine

## Abstract

Materials that are able to switch microwave radiation are strongly desired for their potential applications in electronic devices. In this paper, we show the spin-dependant interaction of spin-crossover materials with microwave radiation, namely, the ability of coordination compounds [Fe(NH_2_trz)_3_]Br_2_ and [Fe(NH_2_trz)_3_](NO_3_)_2_ that undergo a cooperative spin transition between low-spin and high-spin states to operate as thermoswitchable microwave absorbers. The characteristics of the microwave reflection and transmission of these spin-crossover complexes were investigated at variable temperatures. The evolution of both the transmission and reflection spectra in the 26–37 GHz frequency band within the temperature range of spin crossover showed significant differences in the interaction of microwave radiation with the high-spin and low-spin forms of the compounds. The microwave transmission coefficient shows a notable decrease upon transition to the high-spin state, while the reflection coefficient can be both increased or decreased on the characteristic frequencies during the spin transition. The different microwave absorbing properties of the low-spin and high-spin forms are found to be associated with a notable microwave permittivity change upon spin crossover. The switchable microwave reflection/transmission correlates well with the transition characteristics found in the optical and differential scanning calorimetry measurements. These results widen the spectroscopic range in which spin-crossover materials can be applied and contribute to the creation of a preliminary database of the microwave absorbing properties of spin-crossover complexes.

## Abstract 2

Iron(ii) spin-crossover complexes are shown to be effective microwave switches operating near room temprature.

## Graphical abstract

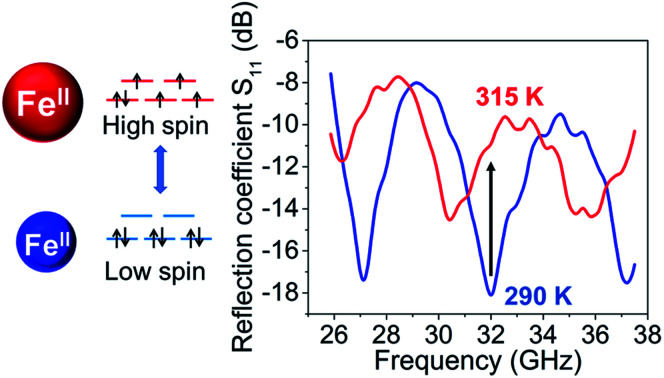

## Introduction

The extensive development of the wireless technology industry has provided new challenges for research during the past decades. At present, among the challenges that involve wireless technologies, there are those that can be solved chemically by the development of new materials: (a) the demand for efficient microwave absorbers; (b) the need of microwave switches that can be tuned to different frequency bands on demand.

Excessive electromagnetic (EM) radiation can compromise the functionality and lifetime of electronic components and can impact human biological systems.^1^ As a result, EM absorbing materials for the microwave frequency range have attracted considerable attention due to their ability to protect certain equipment from external EM pollution.^2^ The interaction of an EM wave with a material is associated with three different processes: the reflection, transmission and absorption of the wave. There are two main criteria for a material to be a good EM absorber: (a) to avoid the reflection of a wave, EM impedance matching between the free space and the absorbing material should be performed; (b) to achieve high absorption of incident waves, the material should display high dielectric and magnetic losses.^3^ A common approach, which is used to fulfil the above-mentioned needs, is the fabrication of composite microwave absorbing materials that consist of magnetic and dielectric loss components. Magnetodielectric core–shell structures of metals and metal-oxides^4^ are very popular along with composites based on graphene^5,6^ and other carbon materials.^7^ Additionally, microwave absorbing polymer composites have been developed to benefit from the light weight nature of such materials.^8^ Different polymers such as epoxy resins, polyurethanes, and various conducting polymers are available as matrices for microwave absorbing composites, which have the advantage of tunable electrical conductivity.^9^ Meanwhile, another huge class of efficient microwave absorbers is represented by metamaterials.^10^

The demand for tunable radio frequency components is associated with the fact that different communication systems such as Wi-Fi, LTE, 5G, and others function in different frequency ranges. Additionally, the standard frequencies of these communication services can somewhat differ in different parts of the world. Consequently, a design of adaptive electronic devices that can be tuned to particular frequency bands requires tunable elements. This is fulfilled by the use of microwave switchable materials that are able, in particular, to tune the microwave transmission and reflection and consequently to block or to allow the passage of waves of certain frequencies under the influence of external stimuli.^11–14^ Notably, switchable radar absorbing materials attract considerable attention.^15^ Except for more classical approaches, which use electrically reconfigurable devices such as PIN diodes^16,17^ or microelectromechanical systems^18,19^ for microwave switching, a new direction that employs phase transition materials to control microwave frequency signal is being developed.^20^ For example, the most studied phase transition material, which was used for microwave switching, is vanadium dioxide (VO_2_). This material is known to undergo a thermally induced metal to insulator transition at ∼68 °C. It has been shown to efficiently switch the microwave signal by both thermal and current activation. There is a possibility with this material to achieve almost complete blocking of a signal in the 1–49 GHz frequency band with 0 dB return loss (complete reflection), 13 to 30 dB insertion loss at room temperature, rather good signal passage with a 10 dB return loss, and a 4 dB insertion loss at a temperature above the phase transition.^13^

In this work, we use a different approach for microwave radiation (MWR) switching by means of a specific class of phase transition materials that undergo a temperature induced spin transition. The coordination compounds of 3d^4^-3d^7^ metals can adopt one of two possible spin states: low-spin (LS) and high-spin (HS). However, some of these compounds are able to undergo spin transitions, namely a change between LS and HS electronic configurations under the influence of various external stimuli, such as a change in temperature^21^ or pressure,^22,23^ light irradiation,^24^ magnetic field^25^ or the presence of guest molecules.^26,27^ Spin crossover (SCO) is accompanied by a drastic change in the magnetic,^28^ optical,^29^ mechanical^30^ and electrical^31^ properties of the materials. These unique abilities of SCO materials improve their application as active elements in chemical sensors,^32^ thermochromic indicators, microthermometers,^33^ mechanical actuators,^30^*etc.*

**Fig. 1:**
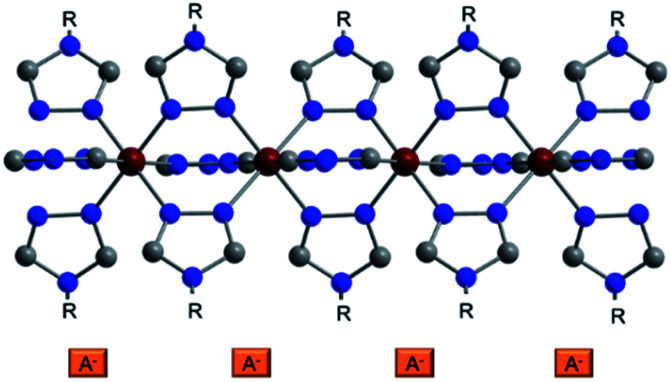
Representation of the crystal structure of a triazolic complex. The anions are depicted schematically; R is for a substituent in the triazole ring. Colour code: Fe dark red, C grey, N blue.

Among all SCO compounds, the complexes based on Fe^II^ and 4-*R*-1,2,4-triazoles^34,35^ form one of the biggest families and are known for the simplicity of their synthesis and an attractive range of transition temperatures. These compounds usually crystallize poorly and their crystal structure has been determined only for two single crystals^36,37^ and some powder samples.^38^ The Fe^2+^ centre in these coordination materials has an octahedral coordination environment [FeN_6_], which is formed by six molecules of 4-*R*-1,2,4-triazole ligands that bind the metallic centres into infinite 1D polymeric chains (Fig. 1). The anions are located in the cavities between the polymeric chains and ensure their linkage into supramolecular 3D architectures. Depending on the substituent of a triazole ligand and counteranion, different transition temperatures, the abruptness of SCO and hysteresis loops can be achieved.

Importantly, the HS and LS forms of spin transition materials tend to interact differently with EM radiation over a wide frequency range. Our group reported recently the ability of a SCO complex [Fe(Htrz)_2_(trz)]BF_4_ (Htrz = 1*H*-1,2,4-triazole, trz = deprotonated triazolato ligand) to interact with MWR differently above and below 377 K.^39^

To develop this further, we targeted SCO switches that can alter the interaction with MWR near room temperature. We closely examined the mechanism responsible for such switchable microwave absorption, which is a spin transition induced change in microwave permittivity. At this stage of research, our primary aim is to develop a database of absorbing and switching properties of SCO complexes in the microwave range towards the future design of efficient microwave switching devices based on SCO materials.

## Results and discussion

### Characterization of SCO properties

Complexes [Fe(NH_2_trz)_3_]Br_2_ (1) and [Fe(NH_2_trz)_3_](NO_3_)_2_ (2) (NH_2_trz = 4-amino-1,2,4-triazole) were chosen due to the temperatures of their spin transitions. These spectacular compounds were first obtained by Lavrenova *et al.* in 1990 and 1986, respectively.^40,41^ Complex 1 has been used for the preparation of nanoparticles^42^ and for the determination of the differential catalytic activity of LS and HS Fe complexes.^44^ Complex 2 has been used for incorporation into optical elements.^33^ Both complexes were investigated by means of polarized Raman scattering.^43^ Notably, both 1 and 2 were previously used to analyse the temperature-dependent dielectric properties of SCO materials in the kHz and THz regions.^45–47^

**Fig. 2:**
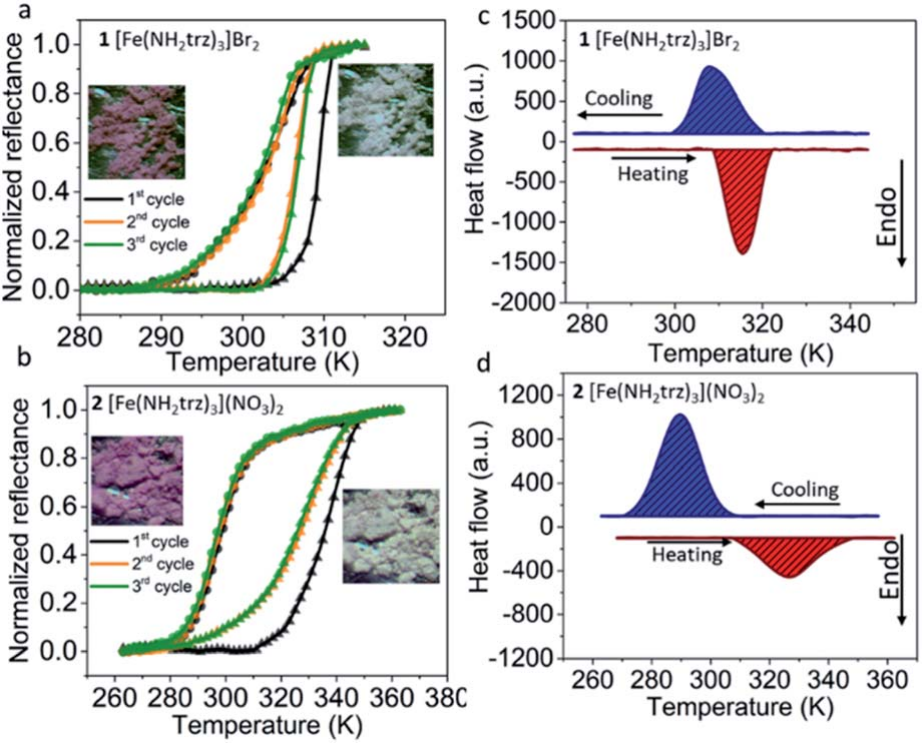
Curves of the thermal spin transition in 1 (a) and 2 (b) obtained by optical reflectance measurements in heating (triangles) and cooling (circles) modes showing a cooperative thermal SCO near room temperature (heating/cooling rate 5 K min^−1^). Insets: images of powders of 1 and 2 in LS and HS states. DSC curves for switchable compounds 1 (c) and 2 (d) showing endothermic/exothermic anomalies of the heat flow caused by thermally induced spin transitions (heating/cooling rates 10 K min^−1^).

The SCO in 1 and 2 has primarily been analysed *via* optical reflectance and differential scanning calorimetry (DSC) measurements. During the SCO, along with the change of electronic configuration (t_2g_^4^e_g_^2^ for HS and t_2g_^6^ for LS), the set of possible electronic transitions for each form of a complex is changed. As a result, this provides a thermochromic effect, which in this case is observed as a distinct change from a pink colour for the LS form to colourless for the HS form (Fig. 2a and b, insets). Consequently, the spin transition in materials can be analysed by temperature dependant optical monitoring due to the presence of a precise colour change.

The reflectance measurements of the above-mentioned complexes in consequent cycles are given in Fig. 2a and b. In both cases, the first cycle slightly differs from the following ones. This effect is frequently observed for triazole-based SCO complexes and may be associated with the loss of residual traces of solvent during the first heating and/or some minor irreversible structural changes. The second and third cycles for compound 1 show that at low temperatures, its reflectance is stable until the temperature of the spin transition is reached.

At the critical temperature *T*_c_↑ = 307 K, an abrupt transition from the LS to the HS state is observed. Upon cooling, the complex switches back in a slightly more gradual manner to the LS state at *T*_c_↓ = 300 K. Different spin transition temperatures in the heating and cooling modes reveals a hysteresis of the spin transition of Δ*T* = 7 K. Upon heating in the second and third cycles, complex 2 undergoes a spin transition at critical temperature *T*_c_↑ = 327 K. Upon cooling, the transition occurs at *T*_c_↓ = 298 K, resulting in a hysteresis loop of Δ*T* = 29 K.

The SCO in these samples was additionally characterized by DSC measurements, which were performed in heating and cooling modes in order to evaluate the entropy and enthalpy change during the spin transition. Compound 1 shows one endothermic peak in the heating mode at *T*_c_↑ = 315 K (Δ*H*↑ = 13.0 kJ mol^−1^, Δ*S*↑ = 41.3 J mol^−1^ K^−1^) (Fig. 2c). Upon cooling, the exothermic peak of the HS → LS transition is observed at *T*_c_↓ = 308 K (Δ*H*↓ = −12.7 kJ mol^−1^, Δ*S*↓ = −41.2 J mol^−1^ K^−1^). Compound 2 shows an endothermic peak in the heating mode at *T*_c_↑ = 326 K (Fig. 2d) (Δ*H*↑ = 10.0 kJ mol^−1^, Δ*S*↑ = 30.7 J mol^−1^ K^−1^). Upon cooling, the transition of 2 occurs at *T*_c_↓ = 290 K (Δ*H*↓ = −15.2 kJ mol^−1^, Δ*S*↓ = −52.4 J mol^−1^ K^−1^). The transition temperatures correlate with those obtained by reflectance measurements and minor differences are due to the thermalization aspects of the experiment caused by different measurement rates, as well as a possible slight shift of the SCO temperature in consequent cycles that can often be observed for Fe^II^ 4-*R*-1,2,4-triazole complexes.

### SCO induced change of microwave reflection/transmission

As was mentioned, the interaction of an EM wave with materials is generally associated with three processes: reflection, absorption and transmission of the EM radiation. The exact behaviour of an EM wave on the surface and in the bulk of a material will depend on its magnetic and electrical properties, which are represented by relative complex permittivity and permeability:

1 *ε* = *ε*′ − i*ε*′′

where *ε* is the complex permittivity with its real *ε*′ and imaginary *ε*′′ parts;

2 *μ* = *μ*′ − i*μ*′′

where *μ* is the complex permeability with its real *μ*′ and imaginary *μ*′′ parts.

*ε*′ is a measure of energy storage from an external electric field in a material and *ε*′′ is responsible for energy dissipation in a medium.

**Fig. 3:**
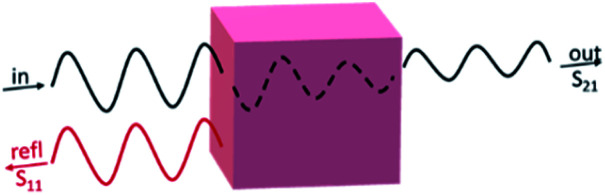
Schematic representation of EM wave transmitted through and reflected back from a sample. Reflection from the output port is not shown.

The flow of the microwave energy through the two-port network can be described by the scattering matrix. Incident microwave signal can be partially reflected after reaching the input port (described by the scattering parameter *S*_11_) and can go inside the port and then exit from the output port (Fig. 3) and be amplified or attenuated (described by the scattering parameter *S*_21_).

In order to fully understand the details of MWR interaction with SCO materials, the EM properties of 1 and 2 were determined by the reflection/transmission method. Pressed powder samples were inserted in a hollow metallic rectangular waveguide with a matched load termination. This means that the incident power is transmitted through or reflected from the sample depending on the material properties. The scalar scattering parameters at variable temperatures were measured using the microwave network analyser in a 26.0–37.5 GHz frequency range. Both samples were heated to 100 °C prior to the measurements for the evaporation of residual traces of solvents. This is to avoid the heating of solvent under the influence of MWR. TGA and IR spectra of 1 and 2 before and after desolvation are given in Fig. S1–S4 (ESI†).

**Fig. 4:**
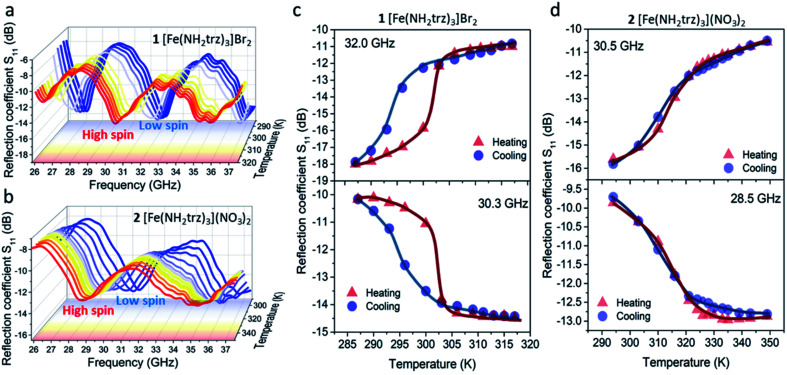
Spectral variation of reflection coefficients for compounds 1 (a) and 2 (b) at different temperatures obtained in the matched waveguide at different temperatures. Reflection coefficient *vs.* temperature for 1 (c) and 2 (d) at selected frequencies. Curves demonstrate the microwave reflection change upon the spin transition.

At room temperature, complex 1 exhibits three reflection bands at 27.1, 32.0, and 37.2 GHz (Fig. 4a). The intensities in these bands reach −17.4, −18.1, and −17.6 dB, respectively. Upon heating with the transition to the HS state, the combination of two effects is observed: a significant shift of the reflection bands *ca.* 1.5 GHz towards lower frequencies and a change of the bands' intensities. As a result, three reflection bands are found at 26.3, 30.4 and 35.8 GHz at the temperatures above the spin transition. The intensities in the newly formed reflection bands at the mentioned frequencies reach −11.8, −14.5 and −14.4 dB, respectively.

Notably, due to the shift of the reflection bands towards lower frequencies during SCO, both the increase and the decrease of reflection can be observed at particular frequencies (Fig. 4c). Consequently, the reflection coefficient of 1 can be tuned by ∼4–8 dB in the 26–37 GHz frequency range with the increase of temperature. Upon further cooling, the *S*_11_ parameter switches back to the initial values at slightly lower temperatures revealing a hysteresis. This in turn causes the effect of “microwave memory”, *i.e.*, the ability to display different microwave properties at the same temperature.

Complex 2 displays similar behavior: two reflection bands at 30.7 and 37.0 GHz can be observed at room temperature (Fig. 4b). The corresponding intensity values reach −15.3 and −15.9 dB. Like 1, a *ca.* 1.5 GHz shift of the reflection bands towards lower frequencies is observed for 2 with its transition to the HS state. In the HS state, reflection bands are located at 28.8 and 35.1 GHz and reach −12.7 and −13.2 dB intensity, respectively. Either the increase or the decrease of reflection by *ca.* 3–5 dB is observed for complex 2 in the 26–37 GHz range (Fig. 4d). The reflection minimum shift towards lower frequencies in both cases is driven by the increase of permittivity caused by the spin transition. Thus, the permittivity change is responsible for such a drastic change in the microwave reflection upon the spin transition.

**Fig. 5:**
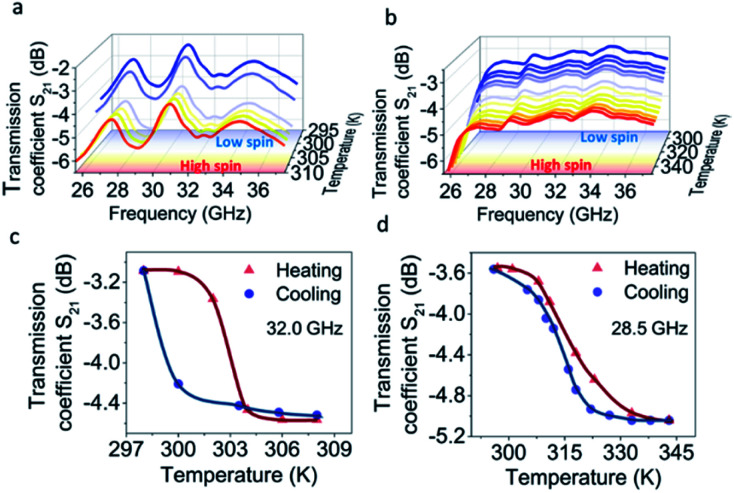
Spectral variation of transmission coefficient of 1 (a) and 2 (b) measured at variable temperatures. Transmission coefficient *vs.* temperature plots of 1 (c) and 2 (d) at selected frequencies. Curves demonstrate a decrease of MWR transmission upon spin transition.

As for the microwave transmission, at lower temperatures the spectra of 1 are characterized by multiple bands. The most prominent ones can be found at 29.0 and 32.8 GHz (Fig. 5a) where the intensities reach −3.89 and −3.35 dB, respectively, when the complex is in the LS state. Those values decrease to −5.44 and −4.90 dB at 302 K with the transition to the HS state, resulting in a 1.55 dB change of the transmission coefficient. After further cooling, the transition back to the LS state occurs at 298 K and the *S*_21_ parameter returns to its initial values (Fig. 5c). Notably, in contrast to the reflection spectra, no shift of the transmission bands towards lower frequencies is observed. A significant frequency shift of the reflection bands is determined by the structure of the measured EM wave.

The measured reflected wave is composed of two waves: one is reflected from the first (input) side of the sample, and the other wave is reflected from the second (output) side. Thus, the second wave passes double the sample length with internal reflection from the second side and obtains a significant phase shift.

In the case of transmission, the phase shift is much smaller as it depends only on the change of the transmission characteristics of the material. Consequently, the frequency shift is not observed.

No meaningful transmission bands are observed for complex 2 in this frequency range. This can be attributed to a higher wave attenuation inside the sample with higher *ε*′′/*ε*′ values. Such attenuation results in the damping of distinct transmission bands. The transmission of −3.5 dB through sample 2 is observed at room temperature (Fig. 5b). This value drops to *ca.* −5 dB with the transition to the HS state at 317 K. The transmission value reverts to the initial number (Fig. 5d) with the reverse transition to the LS state at 313 K. Due to the settle mode of microwave reflection/transmission measurements and the oscillation of the sample temperature, the thermal curves display a smaller hysteresis loop than in the optical reflectance experiments. This effect has a different value for the two complexes, which may be related to the slightly different kinetics of SCO in 1 and 2.

The measured values of reflection and transmission indicate that most of the MWR intensity is absorbed and transmitted, while minor EM power is reflected from the samples. Complexes 1 and 2 have similar capability to switch MWR as they belong to the same class of SCO compounds and most of their physical properties are very similar. At the same time, the differences in reflection and transmission spectra are attributed to the chemical individuality of each compound (different geometries of the samples should also be considered).

A comparison of the reflection and transmission parameters of three spin-crossover complexes (1, 2, [Fe(Htrz)_2_(trz)]BF_4_) and VO_2_ (which is the most known material for microwave switching) is given in Table S2 (ESI†). Although the switching effect is less prominent in the case of SCO complexes compared to VO_2_, SCO materials can offer a wide range of necessary switching temperatures and apparently can be considered as prospective MWR switches, and can also be used as controllable components for microwave devices.

### Dielectric properties of SCO complexes in the microwave frequency domain

**Fig. 6:**
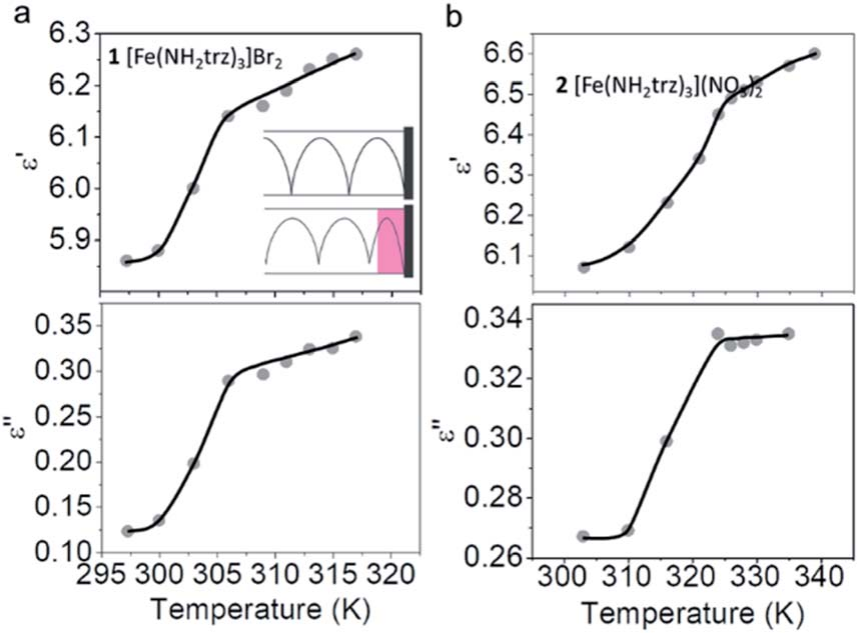
Real and imaginary parts of permittivity *versus* temperature at 37 GHz for 1 (a) and 2 (b) measured upon heating. The plots demonstrate a significant increase of the permittivity upon a spin transition. Inset: schematic representation of short-circuited waveguide experiment.

The complex permittivity of compounds 1 and 2 was measured in the temperature range of the spin transition by the short-circuited waveguide method at a constant frequency of 37 GHz. In this method, the end of a waveguide is blocked by a metallic plate (short-circuit) that reflects back all the incident EM power. This induces the creation of a standing EM wave inside the waveguide. When the dielectric material is inserted in the waveguide close to the metallic plate at the end, a part of incident energy is absorbed by this material and the length of the EM wave changes depending on the material permittivity. This leads to some shift of the position of the microwave electric field minimum in the standing wave and to some change in the standing wave amplitude (Fig. 6, inset). So, one can derive the permittivity of the material by analysing the standing wave parameters.

For non-ferro(ferri)magnetic materials that are characterized by relative permeability values of *ca.* 1, the complex reflection coefficient S_11_ is measured at the front face of the sample in the short-circuited waveguide and can be represented as:^3^3
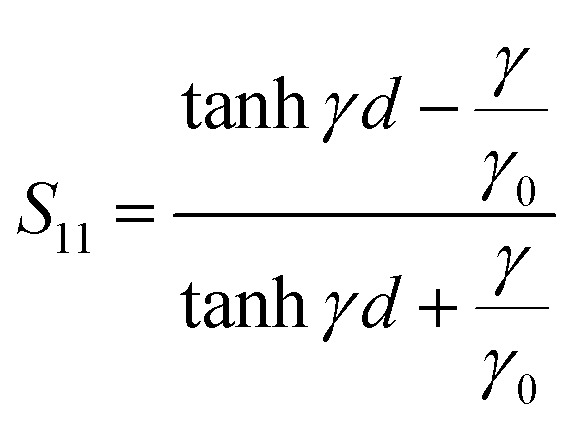
where *γ* and *γ*_0_ are the single-mode EM propagation constants in the sample-filled and air-filled waveguide, respectively, and *d* is the sample thickness.4
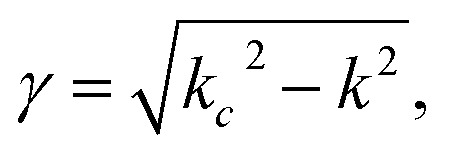
5
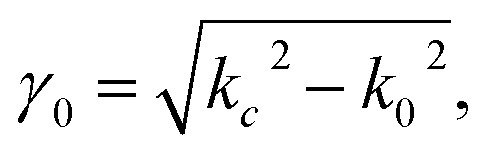
where *k* and *k*_0_ are the wavenumbers that a uniform plane wave would have in a medium-filled space or free space, respectively, and *k*_c_ is the critical EM wavenumber, which depends on the waveguide mode and dimensions.6
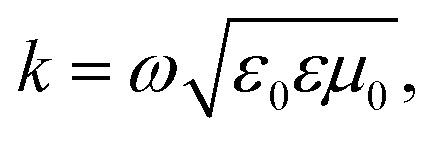
7
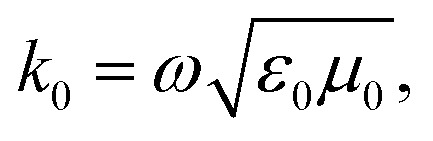
where *ω* is the angular frequency, *ε*_0_ and *μ*_0_ are the permittivity and permeability of free space, respectively, and *ε* is the complex relative permittivity of the material. By solving the complex nonlinear eqn (3), one can obtain the complex EM wavenumber *k*, from which the required complex relative permittivity of a material can be calculated.

Fig. 6a and b display the temperature dependencies of *ε*′ and *ε*′′ for 1 and 2 respectively. The measurements were performed at a constant EM frequency of 37 GHz in heating mode. Heating was performed in a settle mode; namely, prior to measurement, the whole system was thermalized at an exact temperature for *ca.* 10 minutes. The real part of permittivity ε′ for the LS form of complex 1 is 5.86 at room temperature. This value increases with the spin transition on heating and reaches the value of 6.19 for the HS form of 1, resulting in Δ*ε*′ = 0.33. The imaginary part of the permittivity *ε*′′ is estimated at 0.12 for the LS form of 1. *ε*′′ increases upon SCO to the HS state and reaches the value of 0.32. This transition gives rise to an *ε*′′ increase by Δ*ε*′′ = 0.20.

Similar behaviour is observed for 2. At room temperature, while the complex is in the LS state, it has the *ε*′ value of 6.07. This value increases with the thermally induced SCO and reaches 6.57 for the HS form. Consequently, the change of *ε*′ for complex 2 as a result of the spin transition is Δ*ε*' = 0.50. The imaginary part of permittivity for 2 has a value of 0.27 for the LS form. After heating, when complex 2 undergoes the transition to the HS state, this value increases and reaches 0.33 (Δ*ε*′′ = 0.06).

The changes of the real and imaginary parts of permittivity, along with the refraction index and absorption factor of 1, 2 and [Fe(trz)(Htrz)_2_]BF_4_ (ref. 39) are given in Table 1. In all three cases, an increase of all dielectric parameters upon the LS → HS transition is observed. In the case of 1 and 2, the permittivity and its change upon LS → HS are of the same magnitude, however for [Fe(trz)(Htrz)_2_]BF_4_, these values are smaller.

**Table 1:** Comparison of the dielectric properties of 1, 2 and [Fe(Htrz)_2_(trz)]BF_4_ (ref. 39) in LS and HS states

	*ε*′			*ε*′′			Refraction index			Absorption factor (cm^−1^)		
	LS	HS	Δ	LS	HS	Δ	LS	HS	Δ	LS	HS	Δ
[Fe(NH_2_trz)_3_]Br_2_	5.86	6.19	0.33	0.12	0.32	0.20	2.42	2.50	0.08	0.38	0.96	0.58
[Fe(NH_2_trz)_3_](NO_3_)_2_	6.07	6.53	0.46	0.27	0.33	0.06	2.46	2.56	0.10	0.83	1.0	0.17
[Fe(Htrz)_2_(trz)]BF_4_	1.84	2.01	0.17	0.04	0.05	0.01	1.35	1.41	0.06	0.016	0.0172	0.0012

The permittivity change upon the spin transition has been frequently observed for various SCO complexes.^48^ Analysis of the previously reported data shows that usually the permittivity at lower frequencies (such as kHz, GHz and THz) increases upon the LS → HS transition, while at high frequencies (visible region) the permittivity decreases with the transition to the HS state. In general, there is a strong dependence of permittivity on temperature and frequency, which can be described according to the Debye theory: 8
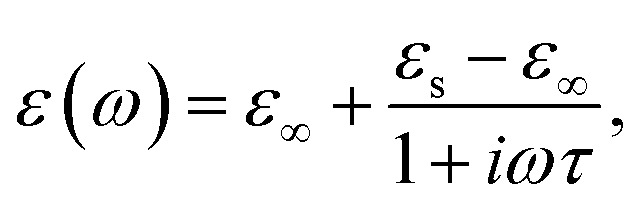
9
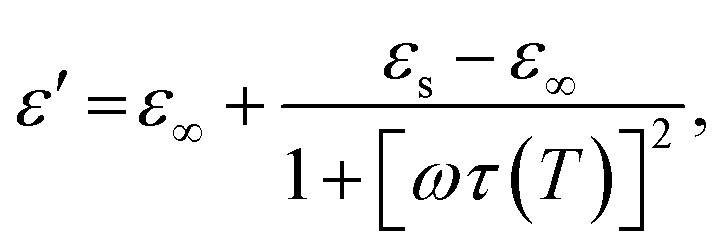
10
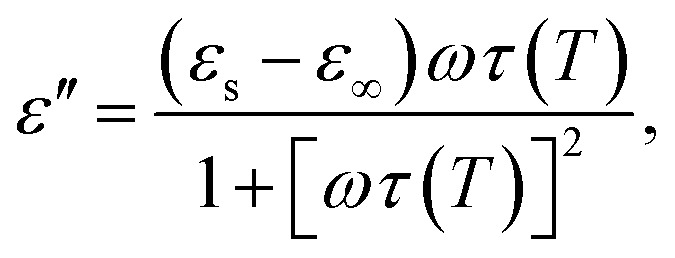
where *ε*_∞_ is the relative permittivity at infinite frequency, *ε*_s_ is the static permittivity, and *τ*(*T*) is the temperature dependent polarization relaxation time.

The permittivity of dielectric materials is determined by their polarizability. Distinct mechanisms of polarizability are realised in different frequency ranges. This leads to the strong frequency dependence of permittivity.

For example, predominantly atomic polarizability is realized in the visible range (around 10^14^ Hz), which is associated with a small deformation of the electron cloud with respect to atom/ion nuclei under the influence of an external electric field. With this type of polarization, the increase of temperature and thermal expansion lead to the decrease of the number of polarized particles in a volume, that in turn causes a decrease of permittivity. Indeed, a decrease of the dielectric constant upon the transition from the LS to the HS state in the visible range for [Fe(NH_2_trz)_3_]Br_2_ has previously been shown.^49,50^

At the same time, at lower frequencies except for atomic polarization, ionic and dipolar mechanisms are realized. These mechanisms have a much stronger influence on the general polarization of a material. The weakening of internal molecular forces with an increase of temperature and volume (in the case of SCO complexes) facilitates the orientation of dipoles, some polar entities such as solvent molecules, and the displacement of ions under the influence of an external electric field. In turn, this effect evokes an increase of polarizability and consequently permittivity upon heating.^45–47,51–54^ The increase of permittivity and other dielectric parameters at lower frequencies (10^3^–10^12^ Hz) upon the transition from the LS to the HS state is a general trend for the majority of SCO complexes, which are usually classic dielectrics.

In this way, the main origin of the permittivity change in SCO materials is the increase of the Fe–N bond length and, consequently, the volume during the LS → HS transition. This expansion leads to the change of polarizability, which directly influences the values of permittivity. Thus, the nature of the permittivity change is similar for all frequencies, however the direction of this change (either increase or decrease) depends on the mechanism of polarization, which is realized at the exact frequency.

At the same time, there are several interesting examples of a decrease of permittivity with the LS → HS transition at 1 kHz that are associated with some unusual electrical properties. For example, such an effect is observed in [Fe(trz)(Htrz)_2_]BF_4_ due to the charge transport properties of this complex.^55,56^

Additionally, as SCO complexes do not show any magnetic ordering, their dielectric constants and microwave absorbing properties depend mostly on the lattice parameters. This allows for the modification of the switching and absorbing characteristics of SCO materials only by slight changes in the chemical composition.

## Conclusions

In summary, we show that SCO complexes of Fe^II^ with 4-amino-1,2,4-triazole can modulate MWR radiation. Due to the experimentally attractive temperatures of the spin transition, the synthesized microwave switching materials can function near room temperature. The change of microwave absorption in these complexes is caused by an abrupt increase of permittivity triggered by the spin transition. Such a permittivity change causes significant changes in the MWR reflection and transmission. Notably, both an increase and decrease of the microwave reflection can be achieved by means of the SCO complexes operating at specific characteristic frequencies. A hysteresis of the spin transition provides “microwave memory” in these materials, which is the ability to display different absorption properties at the same temperature. Additionally, these results make an important contribution to the creation of a preliminary database of microwave absorbing properties of spin-crossover materials.

## Experimental information

### Materials

Iron(ii) bromide, iron(ii) nitrate and 4-amino-1,2,4-triazole were purchased from UkrOrgSyntez Ltd. and used as received.

### Synthesis

Complexes 1 and 2 were obtained by a slightly modified method from Lavrenova *et al.*^40,41^

#### Synthesis of 1

The powder sample of [Fe(NH_2_trz)_3_]Br_2_ was obtained by adding 1.17 g of 4-amino-1,2,4-triazole (13.89 mmol, 3 eq.) in ethanol (10 mL) to 1 g (4.63 mmol, 1 eq.) of FeBr_2_ in water (10 mL). After mixing the two solutions, a pink precipitate appeared immediately. The mixture was allowed to stand for 24 h, then was filtered, washed with ethanol and dried in air. Anal. Calcd. for FeC_6_N_12_H_12_Br_2_ (%): C 15.38, N 35.90, H 2.56; found: C 15.42, N 35.84, H 2.61.

#### Synthesis of 2

The powder sample of [Fe(NH_2_trz)_3_](NO_3_)_2_ was obtained by adding 1.16 g of 4-amino-1,2,4-triazole (13.86 mmol, 3 eq.) in ethanol (5 mL) to 1.58 g (4.62 mmol, 1 eq.) of Fe(NO_3_)_2_·9H_2_O in water (6 mL). After mixing the two solutions, white precipitate appeared immediately and turned pink after *ca.* 2 hours. The mixture was allowed to stand for 24 h, then was filtered off, washed with ethanol and dried in air. Anal. Calcd. for FeC_6_N_14_H_12_O_6_ (%): C 16.67, N 45.37, 2.78; found: C16.72, N 45.31, H 2.72.

### Characterization and measurements

Optical reflectance measurements were conducted using a Linkam DSC 600 temperature-controlled microscope stage cryostat and Optica SZM-1 stereomicroscope equipped with a Sigeta UCMOS 1300 camera. The processing of images was performed with ImageJ software. The measurements were carried out using a green colour filter. Reflectance measurements were performed with a heating/cooling rate of 5 K min^−1^.

Differential scanning calorimetry measurements were performed with a Linkam DSC 600. DSC profiles were recorded with a heating/cooling rate of 10 K min^−1^.

Microwave measurements were carried out with a P2–65 scalar microwave network analyzer operating in the Ka frequency band. An analyzer was equipped with a hollow rectangular waveguide (7.20 × 3.40 mm). The SCO complexes were heated to 100 °C for 10 minutes for the evaporation of residual traces of solvents prior to measurements (TGA is given in Fig. S3 and S4†). The powder samples were pressed into rectangular pellets *in situ* in the waveguide by applying *ca.* 20 MPa pressure (*d* = 8.65 mm). The waveguide filled with SCO complexes was heated with an external thermostat for temperature dependent measurements. Narrow thermal intervals for MW measurements were selected for each complex from the preceding optical and DSC experiments.

Elemental analyses (C, H, N) were performed with a Vario EL III element analyser.

Infrared spectra were recorded with a Bruker Vertex 70 spectrometer using the ATR technique.

Thermogravimetric analysis (TGA) was performed with a Shimadzu DTG-60H simultaneous DTA-TG apparatus.

## Conflicts of interest

There are no conflicts to declare.

## Supplementary Material

RA-010-D0RA02236E-s001
