# Supplementary material for: Tunable microwave absorption of switchable complexes operating near room temperature
Source: RSC Adv. 2020 Jun 5;10(36):21621–8. doi: 10.1039/d0ra02236e (PMC9054383; doi:10.1039/d0ra02236e)
Supplement: RA-010-D0RA02236E-s001 [file RA-010-D0RA02236E-s001.pdf]

Electronic Supplementary Material (ESI) for RSC Advances.
This journal is © The Royal Society of Chemistry 2020

SUPPORTING INFORMATION

Tunable Microwave Absorption of Switchable Complexes Operating Near Room Temperature

Olesia I. Kucheriv,[a] *Viktor V. Oliynyk,*[b] *Volodymyr V. Zagorodnii,*[b] *Vilen L. Launets,*[b] *Olena V. Penkivska,*[b] *Igor O. Fritsky,*[a] *and Il'ya A. Gural'skiy* [a*]

[a]Department of Chemistry, Taras Shevchenko National University of Kyiv, 64 Volodymyrska St., Kyiv 01601, Ukraine

[b]Department of Radio Physics, Electronics and Computer Systems, Taras Shevchenko National University of Kyiv, 64 Volodymyrska St., Kyiv 01601, Ukraine

Contents

Figure S1. IR spectrum of **1.** **S2**

Figure S2. IR spectrum of **2.** **S2**

Figure S3. TGA of **1.** **S3**

Figure S4. TGA of **2.** **S3**

Table S1. Interpretation of IR bands of **1** and **2**. **S4**

Table S2. Comparison of microwave reflection/transmission parameters of **1**, **2**, $[Fe(Htrz)_2(trz)]BF_4$ and VO_2. **S4**

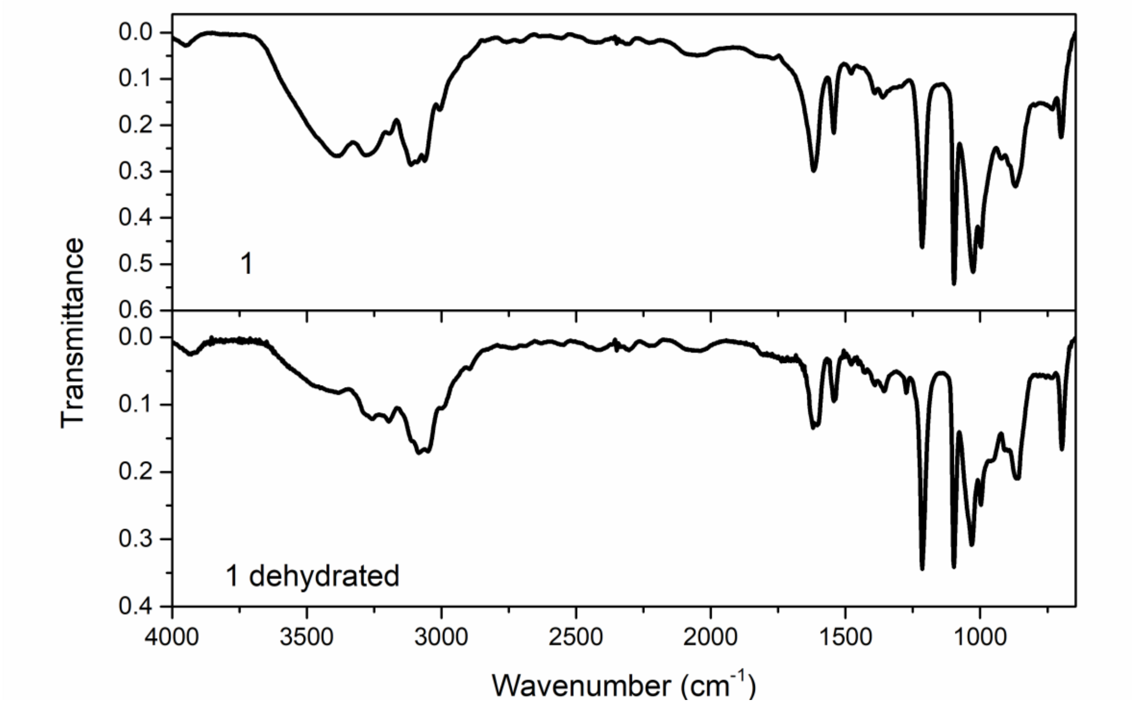

Figure S1. IR spectrum of as received (top) and dehydrated (down) sample of **1** recorded using ATR technique at room temperature**.**

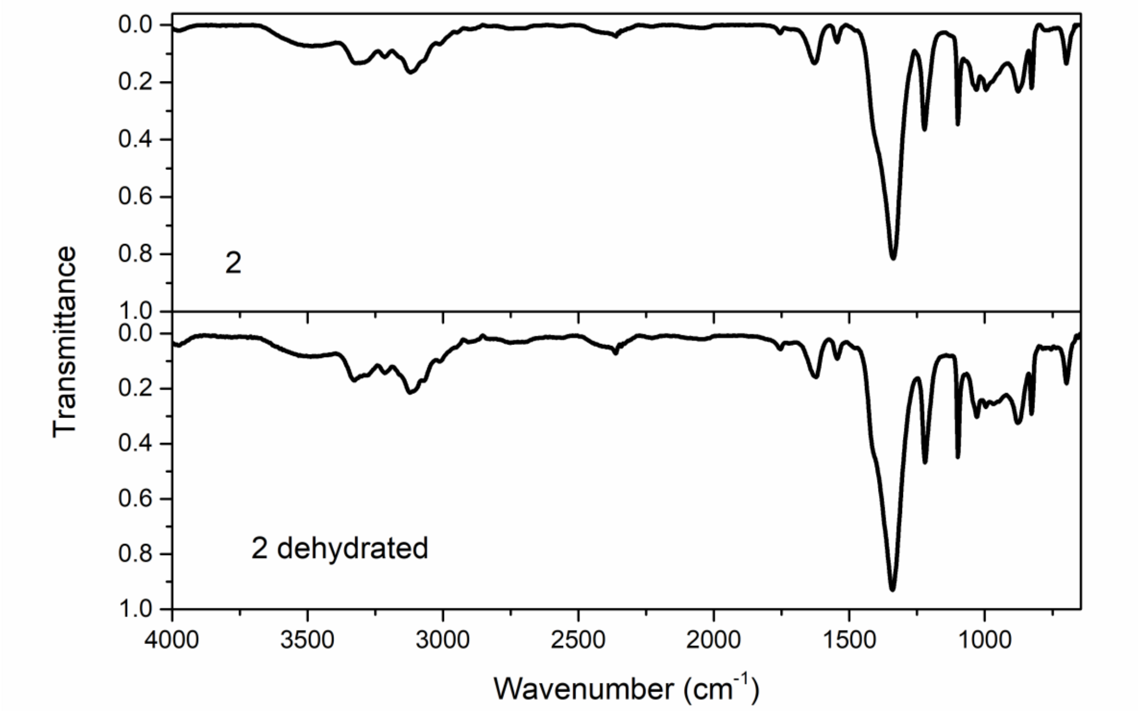

Figure S2. IR spectrum of as received (top) and dehydrated (down) sample of **2** recorded using ATR technique at room temperature**.**

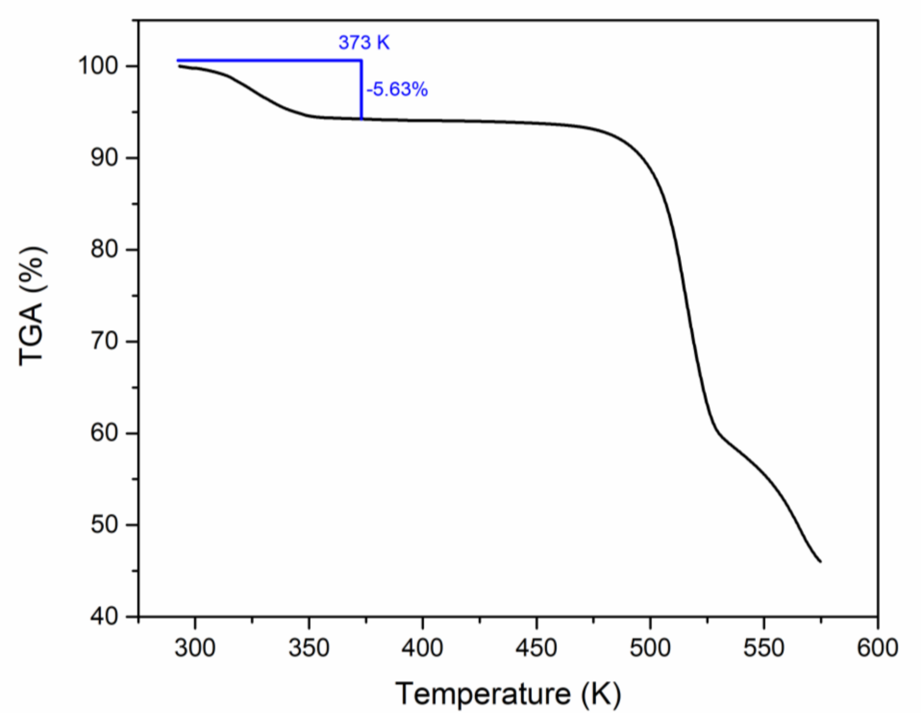

Figure S3. TGA of **1** showing the loss of 5.63% of mass up to 100 °C due to evaporation of residual traces of solvents**.**

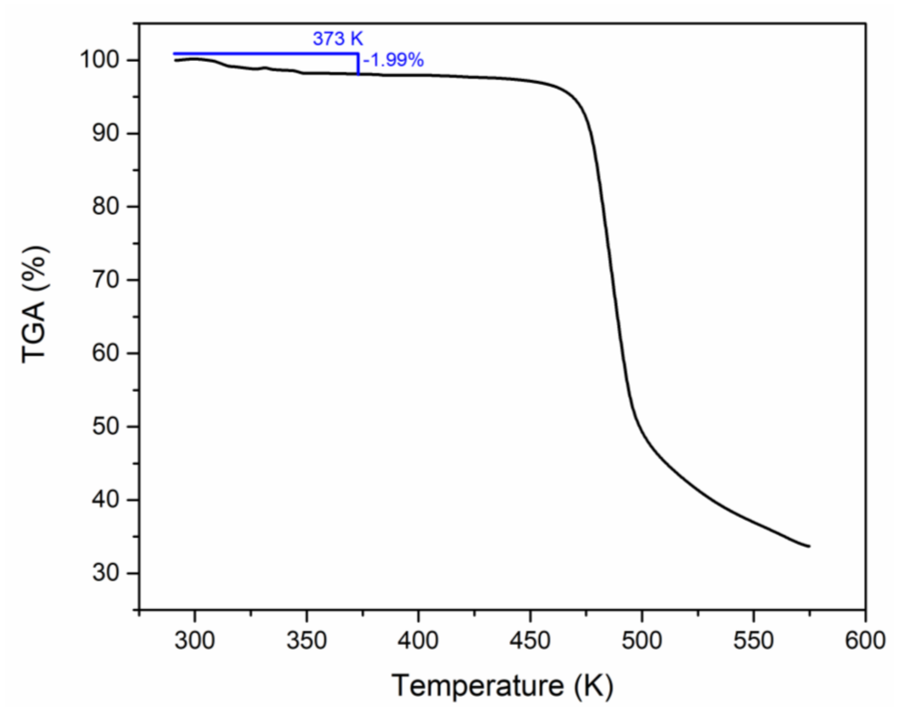

Figure S4. TGA of **2** showing the loss of 1.99% of mass up to 100 °C due to evaporation of residual traces of solvents**.**

Table S1. Interpretation of IR bands of **1** and **2**.

	**1** $[Fe(NH_2trz)_3]Br_2$	**2** $[Fe(NH_2trz)_3](NO_3)_2$
Ring deformation	690	697
$\omega(NH_2)$	867	821
Ring deformation	995, 1026	994, 1030
δ(CH)	1096	1099
$\nu(N\text{-}NH_2)$	1215	1219
ν(NO)	-	1336
Ring deformation	1479, 1543	1545
$\delta(NH_2)$	1619	1626
$\nu(NH_2)$, ν(CH), ν(OH) of water molecules	3000-3500	3000-3500

ν – stretching, δ – bending (scissoring), ω – out-of-plane bending

Table S2. Comparison of microwave reflection/transmission parameters of **1**, **2**, $[Fe(Htrz)_2(trz)]BF_4$[1] and VO_2.[2]

	$[Fe(NH_2trz)_3]Br_2$ 32 GHz		$[Fe(NH_2trz)_3](NO_3)_2$ 28.5 GHz		$[Fe(Htrz)_2(trz)]BF_4$ 27 GHz		VO_2* 30 GHz	
	LS	HS	LS	HS	LS	HS	LT	HT
S_{11} (dB)	-17.9	-10.8	-9.7	-12.9	Not measured		-1	-10
S_{21} (dB)	-3.1	-4.6	-3.6	-5.0	-0.9	-1.3	-15	-4

LT – low temperature (below phase transition)
HT – high temperature (above phase transition)
* Values of S_{11} and S_{21} for VO_2 are deduced from the figure in ref. 2.

(1) Kucheriv, O. I.; Oliynyk, V. V.; Zagorodnii, V. V.; Launets, V. L.; Gural'skiy, I. A. Spin-Crossover Materials towards Microwave Radiation Switches. *Sci. Rep.* **2016**, *6* (1), 38334.

(2) Yang, S.; Vaseem, M.; Shamim, A. Fully Inkjet-Printed VO_2 -Based Radio-Frequency Switches for Flexible Reconfigurable Components. *Adv. Mater. Technol.* **2019**, *4* (1), 1800276.
